# Proximity Interactions among Basal Body Components in *Trypanosoma brucei* Identify Novel Regulators of Basal Body Biogenesis and Inheritance

**DOI:** 10.1128/mBio.02120-16

**Published:** 2017-01-03

**Authors:** Hung Quang Dang, Qing Zhou, Veronica W. Rowlett, Huiqing Hu, Kyu Joon Lee, William Margolin, Ziyin Li

**Affiliations:** Department of Microbiology and Molecular Genetics, McGovern Medical School, University of Texas Health Science Center at Houston, Houston, Texas, USA; Washington University School of Medicine

## Abstract

The basal body shares similar architecture with centrioles in animals and is involved in nucleating flagellar axonemal microtubules in flagellated eukaryotes. The early-branching *Trypanosoma brucei* possesses a motile flagellum nucleated from the basal body that consists of a mature basal body and an adjacent pro-basal body. Little is known about the basal body proteome and its roles in basal body biogenesis and flagellar axoneme assembly in *T. brucei*. Here, we report the identification of 14 conserved centriole/basal body protein homologs and 25 trypanosome-specific basal body proteins. These proteins localize to distinct subdomains of the basal body, and several of them form a ring-like structure surrounding the basal body barrel. Functional characterization of representative basal body proteins revealed distinct roles in basal body duplication/separation and flagellar axoneme assembly. Overall, this work identified novel proteins required for basal body duplication and separation and uncovered new functions of conserved basal body proteins in basal body duplication and separation, highlighting an unusual mechanism of basal body biogenesis and inheritance in this early divergent eukaryote.

## IMPORTANCE

The basal body in the early-branching protozoan *Trypanosoma brucei* nucleates flagellum assembly and also regulates organelle segregation, cell morphogenesis, and cell division. However, the molecular composition and the assembly process of the basal body remain poorly understood. Here, we identify 14 conserved basal body proteins and 25 trypanosome-specific basal body proteins via bioinformatics, localization-based screening, and proximity-dependent biotin identification. We further localized these proteins to distinct subdomains of the basal body by using fluorescence microscopy and superresolution microscopy, discovered novel regulators of basal body duplication and separation, and uncovered new functions of conserved basal body proteins in basal body duplication and separation. This work lays the foundation for dissecting the mechanisms underlying basal body biogenesis and inheritance in *T. brucei*.

## INTRODUCTION

The basal body in flagellated organisms is structurally similar to the centriole in animals and is characterized by a 9-fold array of microtubule triplets surrounding the cartwheel structure that is located in the proximal end of the basal body. The cartwheel is built by nine homodimers of SAS-6 protein and connects to the A-microtubules of the microtubule triplets through the pinhead structure made by the BLD10/CEP135 protein (1, 2). SAS-6 and BLD10/CEP135 are among the 14 ancestral centriole core components that have been identified in 45 organisms (3) and, together with SAS-4/CPAP, they are considered the core ancestral module involved in centriole/basal body biogenesis (4). While SAS-6 and BLD10/CEP135 form the cartwheel structure of the centriole and the basal body, SAS-4/CPAP is required for elongation and stabilization of centriolar microtubules (5, 6).

*Trypanosoma brucei*, an early divergent protozoan parasite, possesses a motile flagellum composed of a canonical 9 + 2 microtubule axoneme and a paraflagellar rod (7). The flagellum is assembled from the basal body, one of the cell’s microtubule organizing centers (MTOCs). The basal body in a G_1_-phase cell comprises a mature basal body (mBB), which nucleates the flagellar axoneme, and an adjacent pro-basal body (pBB) which, upon entry into the S phase of the cell cycle, nucleates the assembly of a new flagellum and thus becomes a new mBB (8). Subsequently, two new pBBs are assembled next to the two mBBS, and one mBB/pBB pair moves to the posterior region of the cell (9, 10). *T. brucei* contains the evolutionarily conserved SAS-4 and SAS-6 homologs (11, 12) and a highly divergent BLD10 homolog (4). While TbSAS-6 is functionally conserved (11), TbSAS-4 is not localized to the basal body and plays a distinct function in life cycle transitions (12), and TbBLD10 has not been experimentally confirmed as a bona fide component of the basal body. Additionally, the *T. brucei* genome also encodes the homologs of several of the 14 ancestral centriole proteins (3), among which only TbCentrin2 (13), TbSAS-6 (11), and WDR16 (3) were confirmed as basal body components; intriguingly, TbDIP13 does not localize to the basal body (14). Strikingly, *T. brucei* appears to lack many conserved basal body protein homologs (3, 4) and does not employ the conserved polo-like kinase-mediated signaling pathway to govern basal body biogenesis (11, 15). These findings suggest an unusual mechanism for basal body duplication in *T. brucei* and also necessitate further exploration of basal body proteome and discovery of trypanosome-specific regulators.

In this report, we carried out bioinformatics analysis to identify the evolutionarily conserved centriole/basal body protein homologs in *T. brucei* and performed proximity-dependent biotin identification (BioID) (16) and subcellular localization-based screening to identify trypanosome-specific basal body proteins. These collective efforts allowed us to identify 14 conserved centriole/basal body protein homologs and 25 trypanosome-specific basal body proteins. Functional characterization of representative basal body proteins uncovered their essential roles in basal body duplication/separation and flagellar axoneme assembly. This work represents a major step forward toward the determination of *T. brucei* basal body proteome and the understanding of basal body duplication, and it highlights the essential involvement of trypanosome-specific proteins in regulating basal body duplication and separation.

## RESULTS

### Identification of basal body proteins in *T. brucei.*

Although a number of evolutionarily conserved centriole/basal body protein homologs have been identified in *T. brucei* by bioinformatics analyses (3, 4), many basal body proteins from *Chlamydomonas reinhardtii* and centrosome proteins from humans have not been used to search for *T. brucei* homologs. As our first effort toward the identification of the *T. brucei* basal body proteome, we queried the *T. brucei* proteome with all *Chlamydomonas* basal body proteins and human centrosome proteins. These analyses allowed us to confirm all of the previously reported homologs (3, 4), including TbCEP76, TbCEP164, TbPOC1, TbPOC5, TbDIP13, and TbBLD10 (see Table S1 in the supplemental material), and additionally allowed the identification of five new homologs, TbCEP19, TbCEP44, TbCEP57, TbCEP120, and TbPOC11 (Table S1). TbCEP164 has three paralogs (Tb927.5.2440, Tb927.11.11650, and Tb927.1.3560) which were named TbCEP164A, TbCEP164B, and TbCEP164C, respectively. TbCEP120 has two paralogs (Tb927.7.6250 and Tb927.11.8920), and these were named TbCEP120A and TbCEP120B, respectively.

10.1128/mBio.02120-16.7 TABLE S1  Conserved and novel basal body proteins of *T. brucei*. Download Table S1, PDF file, 0.1 MB. Copyright © 2017 Dang et al. 2017 Dang et al. This content is distributed under the terms of the Creative Commons Attribution 4.0 International license.

To identify novel basal body proteins in *T. brucei*, we carried out BioID using TbSAS-6, TbCEP57, and TbPOC11 as the baits. These proteins were chosen because our RNA interference (RNAi) data showed that they were all essential for cell viability (11) (see below). Tetracycline-induced expression of BirA*-hemaglgutinin (HA) fusion proteins was confirmed by Western blotting (Fig. S1A, D, and G), and localization of the fusion proteins to the basal body was confirmed by immunofluorescence microscopy (Fig. S1B, E, and H). Overexpression of BirA* fusion proteins did not affect cell growth (data not shown). Biotinylated proteins were purified from both the cytosolic and cytoskeletal fractions of both the control and tetracycline-induced cells (Fig. S1C, F, and I), digested with trypsin, analyzed by liquid chromatography-tandem mass spectrometry (LC-MS/MS), and searched against the *T. brucei* proteome. By comparing the protein hits between the control cells and tetracycline-induced cells, nonspecific proteins that were detected in the control cells were removed.

10.1128/mBio.02120-16.1 FIGURE S1  Identification of binding partners and near neighbors of TbSAS-6, TbPOC11, TbCEP57, and TbBBP46 by BioID. (A, D, G, and J) Western blotting to detect the expression of BirA-3HA-fused TbSAS-6 (A), TbPOC11 (D), TbCEP57 (G), and TbBBP46 (J). TbPSA6 served as the loading control. (B, E, H, and K) Immunofluorescence microscopic examination of the localization of BirA-3HA-fused TbSAS-6 (B), TbPOC11 (E), TbCEP57 (H), and TbBBP46 (K). Bar, 5 µm. (C, F, I, and L) Affinity purification of biotinylated proteins from cells expressing BirA-3HA-fused TbSAS-6 (C), TbPOC11 (F), TbCEP57 (I), and TbBBP46 (L). The noninduced cells served as the control. Download Figure S1, PDF file, 0.4 MB. Copyright © 2017 Dang et al. 2017 Dang et al. This content is distributed under the terms of the Creative Commons Attribution 4.0 International license.

We then searched the protein hits for known trypanosome basal body proteins and conserved centriole/basal body protein homologs. BioID with TbSAS-6 as the bait identified KMP-11, a known basal body protein (17), and two centriole/basal body protein homologs, TbPOC1 and TbBLD10 (Fig. S2). TbPOC11 BioID identified four known basal body proteins, KMP-11, TBBC, TbCentrin2, and TbCentrin4, and four centriole/basal body protein homologs, TbSAS-6, TbPOC1, TbCEP164B, and TbBLD10 (Fig. S2). BioID with TbCEP57 as the bait identified two known basal body proteins, SPBB1 (18) and TBCCD1 (19), and one centriole/basal body protein homolog, TbCEP120B (Fig. S2).

10.1128/mBio.02120-16.2 FIGURE S2  Proximity-based interaction map of *T. brucei* basal body proteins. BioID was carried out with TbSAS-6, TbPOC11, TbCEP57, and TbBBP46 as baits. Blue lines indicate the proximity interactions detected with TbSAS-6 BioID, black lines show the proximity interactions detected by TbPOC11 BioID, green lines indicate the proximity interactions detected by TbCEP57 BioID, and pink lines show the proximity interactions detected by TbBBP46 BioID. Download Figure S2, PDF file, 0.1 MB. Copyright © 2017 Dang et al. 2017 Dang et al. This content is distributed under the terms of the Creative Commons Attribution 4.0 International license.

To identify trypanosome-specific basal body proteins, the hypothetical proteins that were among the top protein hits from each of the three BioID experiments were endogenously tagged with a triple-HA epitope, and their subcellular localization was determined by immunofluorescence microscopy. These efforts identified a total of 19 trypanosome-specific basal body proteins, among which 7 proteins were identified by both TbPOC11 and TbCEP57 BioID, 6 proteins were identified by TbPOC11 BioID, 5 proteins by TbCEP57 BioID, and 1 protein by both TbSAS-6 and TbPOC11 BioID (Table S1; Fig. S2). We named these proteins TbBBPs for *T. brucei* basal body proteins of XX mass (XX denotes the mass in kilodaltons). TbBBP87, which was detected by TbCEP57 BioID, has a close paralog (Tb927.8.4580). Epitope tagging showed that Tb927.8.4580 also localized to the basal body, and thus we named it TbBBP59. TbBBP46, which was identified by TbCEP57 BioID and was shown to be essential (see below), was further used as the bait for BioID (Fig. S1J to L). TbBBP46 BioID detected γ-tubulin, TbPOC1, TbPOC11, and 6 TbBBPs that were also detected by TbPOC11 (Fig. S2).

We recently also initiated a large-scale protein-tagging project and attempted to identify novel basal body proteins. Among our initial epitope tagging of 159 hypothetical proteins, 5 of them localized to the basal body, TbBBP38, TbBBP52, TbBBP68, TbBBP69, and TbBBP96 (Table S1). In summary, through bioinformatics, BioID, and localization-based screening, a total of 25 trypanosome-specific basal body proteins, including the 19 proteins identified by BioID and the TbBBP87 paralog (TbBBP59), were identified in the current work.

### Trypanosome basal body proteins localize to distinct subdomains of the basal body.

**FIG 1:**
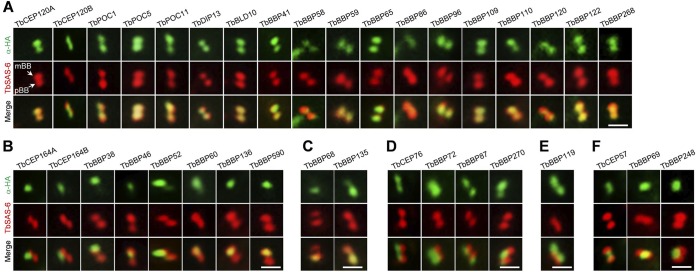
Subcellular localizations of basal body proteins. Proteins were endogenously tagged with a triple-HA epitope and detected by coimmunostaining with FITC-conjugated anti-HA MAb and anti-TbSAS-6 polyclonal antibody. (A) Proteins localizing to the vicinity of mBB and pBB. (B) Proteins localizing to the vicinity of mBB. (C) Proteins localizing to the vicinity of pBB. (D) Proteins localizing to the distal ends (flagellar sides) of mBB and pBB. (E) A protein that localizes to the proximal ends (kinetoplast side) of mBB and pBB. (F) Proteins localizing between mBB and pBB. Bars, 5 µm.

Eleven of the fourteen conserved basal body proteins, except TbCEP19, TbCEP44, and TbCEP164C, and the 25 trypanosome-specific basal body proteins were successfully tagged with a C-terminal triple-HA epitope at the endogenous locus. Coimmunostaining with anti-HA antibody and anti-TbSAS-6 antibody showed that these proteins localized to distinct regions in the basal body. Based on their localizations, these proteins were classified into six groups. The largest group, consisting of 7 conserved basal body proteins and 11 trypanosome-specific proteins, localized to the vicinity of mBB and pBB (Fig. 1A; Fig. S3 and S4). It should be noted that TbBBP58 additionally localized to the flagellum and TbBBP86 additionally was detected at the bilobe structure (Fig. S4). The second largest group, consisting of two CEP164 homologs (TbCEP164A and TbCEP164B) and six trypanosome-specific proteins, localized to the vicinity of mBB (Fig. 1B; Fig. S3 and S4). It was noted that TbCEP164A, TbCEP164B, and TbBBP52 localized to the distal end of mBB, and a small amount of TbBBP52 and TbBBP60 also localized to pBB. The third group, consisting of two proteins (TbBBP68 and TbBBP135), localized primarily to pBB, although a small amount of TbBBP135 was also detected at mBB (Fig. 1C; Fig. S4). The fourth group, consisting of TbCEP76 and three trypanosome-specific proteins, localized to the distal ends of mBB and pBB (Fig. 1D; Fig. S3 and S4), and the fifth group, consisting of only one protein (TbBBP119), localized to the proximal ends of mBB and pBB (Fig. 1E; Fig. S4). The last group, consisting of TbCEP57 and two trypanosome-specific proteins, localized between mBB and pBB (Fig. 1F; Fig. S3 and S4), suggesting that they may act to connect mBB and pBB.

10.1128/mBio.02120-16.3 FIGURE S3  Subcellular localization of the 11 evolutionarily conserved basal body proteins. Each of these proteins was endogenously tagged with a triple-HA epitope at the C terminus. Cells were immunostained with FITC-conjugated anti-HA antibody and TbSAS-6 polyclonal antibody. Bar, 5 µm. Download Figure S3, PDF file, 0.6 MB. Copyright © 2017 Dang et al. 2017 Dang et al. This content is distributed under the terms of the Creative Commons Attribution 4.0 International license.

10.1128/mBio.02120-16.4 FIGURE S4  Subcellular localization of the 25 trypanosome-specific basal body proteins. Each of these proteins was endogenously tagged with a triple-HA epitope at the C terminus. Cells were immunostained with FITC-conjugated anti-HA antibody and TbSAS-6 polyclonal antibody. Bar, 5 µm. Download Figure S4, PDF file, 1.2 MB. Copyright © 2017 Dang et al. 2017 Dang et al. This content is distributed under the terms of the Creative Commons Attribution 4.0 International license.

**FIG 2:**
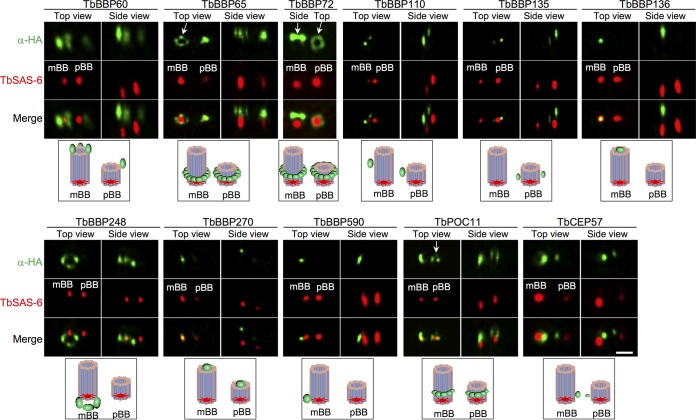
3D-SIM superresolution microscopic analysis of basal body protein localization. Cells were coimmunostained with anti-HA MAb and anti-TbSAS-6 polyclonal antibody and visualized using the DeltaVision OMX v 4 Blaze microscope. The cartoon below the microscopy images illustrates the localization of the protein (in green) in the basal body. About 40 cells were imaged for each protein. Note that the different sizes of mBBs and pBBs in some images could be due to the differences in their distances from the camera. Bars, 0.5 µm.

We next employed three-dimensional structured illumination microscopy (3D-SIM) to visualize at higher resolution the localization of basal body proteins relative to the basal body cartwheel. Among the 11 basal body proteins analyzed, several proteins form a ring-like structure. TbBBP65 and TbBBP72 localized in patches in a ring structure located at the distal region of the cartwheels of mBB and pBB, whereas TbBBP248 localized in patches in a ring-like structure at the mBB cartwheel (Fig. 2). TbBBP60 was detected as three foci at the distal region of the mBB cartwheel and also showed a weak signal near pBB (Fig. 2). TbBBP110 and TbBBP270 were detected as two foci that are located at the distal region of the cartwheels of mBB and pBB, respectively (Fig. 2). However, TbBBP110 localized to the side of the basal body microtubule array, whereas TbBBP270 appeared to localize to the top of the microtubule array (Fig. 2). TbBBP136 and TbBBP590 were both detected as a single focus near or associating with mBB, but TbBBP136 appeared to localize to the distal part of the cartwheel and TbBBP590 appeared to localize to the side of the cartwheel (Fig. 2). TbBBP135 was detected as two foci at the distal region of the pBB cartwheel (Fig. 2). TbPOC11 appeared to form a half-ring structure at the distal region of mBB and pBB cartwheels, whereas TbCEP57 was detected as two foci between mBB and pBB (Fig. 2). These analyses confirmed that these proteins localize to distinct subdomains of the basal body, where they may play distinct roles.

To understand the physiological roles of these basal body proteins in *T. brucei*, we decided to characterize some of the conserved basal body proteins and trypanosome-specific basal body proteins based on their subcellular localizations. We selected TbPOC1, TbPOC11, TbBLD10, TbBBP65 (Fig. 1A), TbBBP46 (Fig. 1B), TbBBP135 (Fig. 1C), TbBBP72 (Fig. 1D), TbBBP119 (Fig. 1E), and TbCEP57 (Fig. 1F) for functional analysis. RNAi of TbPOC11, TbBLD10, TbBBP65, TbBBP46, and TbCEP57, but not of TbPOC1, TbBBP72, and TbBBP119, resulted in growth defects and therefore they were further characterized.

### A divergent BLD10 homolog is required for pBB biogenesis and axoneme assembly.

**FIG 3:**
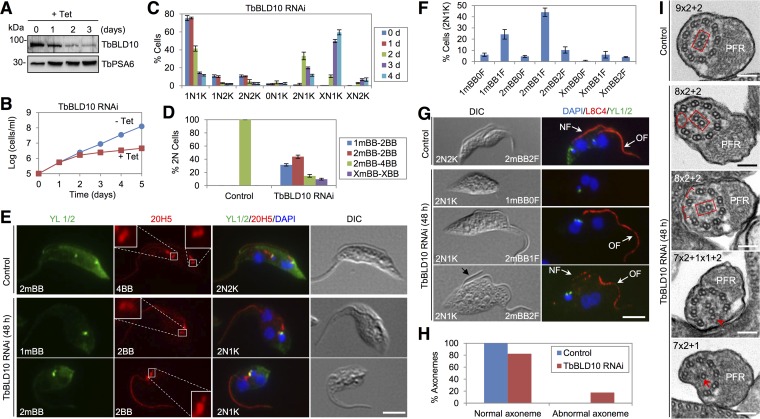
TbBLD10 is required for pro-basal body biogenesis and axoneme assembly. (A) Western blotting with anti-TbBLD10 antibody to monitor the TbBLD10 protein level. Levels of TbPSA6, the *T. brucei* proteasome subunit alpha-6, served as the loading control. (B) RNAi of TbBLD10 inhibited cell proliferation. (C) Quantification of the numbers of nuclei (N) and kinetoplasts (K) before and after TbBLD10 RNAi. A total of 200 cells were counted for each time point, and error bars indicate standard deviations calculated from three independent experiments. (D) Quantification of cells with different numbers of mBBs and total basal body (mBBs and pBBs) in control 2N2K cells and TbBLD10-deficient 2N1K cells. A total of 200 cells were counted for each cell type, and error bars indicate standard deviations calculated from three independent experiments. (E) Coimmunostaining of cells with YL 1/2 to label mBBs and with 20H5 to label mBBs and pBBs. Bar, 5 µm. (F) Quantification of cells with different numbers of flagella and mBBs in TbBLD10-deficient 2N1K cells. A total of 200 cells were counted, and error bars indicate standard deviations calculated from three independent experiments. (G) Coimmunostaining of cells with L8C4 to label the flagella and YL 1/2 to label mBBs. NF, new flagellum; OF, old flagellum. Bar, 5 µm. (H) Quantification of axonemes with normal or abnormal structures in control and TbBLD10 RNAi cells. A total of 95 sections from control cells and 126 sections from TbBLD10 RNAi cells were counted. (I) Morphology of the axoneme in control and TbBLD10 RNAi cells. Note that the orientation of the central pair (outlined in a red rectangle) in TbBLD10 RNAi cells was altered. The red oval outlines the missing outer doublet in the axoneme of a TbBLD10 RNAi cell. The red brackets show the enlarged distance between the outer doublets in a TbBLD10 RNAi cell. The red open arrowhead indicates an outer singlet in a TbBLD10 RNAi cell. The red arrow indicates a central singlet, instead of a central pair, in a TbBLD10 RNAi cell. Bars, 100 nm.

TbBLD10 was identified by bioinformatics and TbSAS-6 BioID (Fig. S2). It is considerably smaller than HsCEP135/BLD10 and CrBLD10, lacking the N-terminal CEP135/BLD10 conserved domain (Fig. S5A) but containing a C-terminal CEP135/BLD10 conserved domain (Fig. S5A and B). To investigate the function of TbBLD10, RNAi was carried out, which resulted in a gradual decrease of TbBLD10 protein as monitored by Western blotting with anti-TbBLD10 antibody (Fig. 3A). RNAi of TbBLD10 caused severe growth inhibition and eventual cell death (Fig. 3B), suggesting that TbBLD10 is essential for cell viability. TbBLD10 knockdown resulted in an initial accumulation of cells with two nuclei and one kinetoplast (2N1K) and subsequent emergence of cells with multiple (>2) nuclei and one kinetoplast (XN1K, X > 2) (Fig. 3C), indicating that kinetoplast segregation was inhibited. Since kinetoplast segregation is mediated by basal body separation, we investigated the potential defects on basal body duplication by coimmunostaining the cells with YL 1/2, which labels mBB, and 20H5, which stains mBB and pBB. We found that the majority (~75%) of the 2N1K cells contained either one mBB and two basal bodies (1mBB-2BB) or two mBBs/two basal bodies (2mBB-2BB) (Fig. 3D and E), indicating defective biogenesis of the new pBB.

10.1128/mBio.02120-16.5 FIGURE S5  *T. brucei* expresses a highly divergent BLD10 homolog. (A) Schematic drawing of the conserved domains in TbBLD10 and the human BLD10 homolog HsCEP135. (B) Sequence alignment of the BLD10/CEP135 conserved region among Chlamydomonas reinhardtii BLD10 (CrBLD10), human CEP135, and TbBLD10. Identical residues are highlighted in red and indicated by asterisks, whereas homologous residues are highlighted in green and indicated by colons or periods. (C and D) TbBLD10 RNAi produced 1N1K cells with a short flagellum or no flagellum. (C) Immunostaining of 1N1K cells from control and TbBLD10 RNAi (48 h) with L8C4 and YL 1/2. Bar, 5 µm. (D) Quantification of 1N1K cells with different numbers of mature basal body and flagella from control and TbBLD10 RNAi (48 h). mBB, mature basal body; sF, short flagellum. Error bars indicate standard deviations. Download Figure S5, PDF file, 0.3 MB. Copyright © 2017 Dang et al. 2017 Dang et al. This content is distributed under the terms of the Creative Commons Attribution 4.0 International license.

We next examined the effect of TbBLD10 RNAi on flagellum assembly by immunostaining the cells with L8C4. The results showed that the majority (~74%) of the 2N1K cells contained a single flagellum (Fig. 3F and G), and the rest of the 2N1K cells contained either two flagella (~14%), one of which was short (Fig. 3G, black arrow), or no flagellum (~11%) (Fig. 3F and G). TbBLD10 RNAi also produced small-sized 1N1K cells with a short flagellum or no flagellum (Fig. S5C and D), which likely were derived from the division of the 2N2K cells with a short, new flagellum or without the new flagellum. These results suggest that TbBLD10 deficiency disrupts new flagellum assembly.

Transmission electron microscopy (TEM) was performed to examine the potential defects in the flagellar axoneme structure. About 17% of the flagellum sections of the TbBLD10 RNAi cells exhibited an abnormal axoneme structure (Fig. 3H). A variety of abnormal axoneme structures was observed, with the majority of them missing one outer doublet and occasionally containing a misoriented central pair (Fig. 3I). In some of the 8×2 + 2 axonemes, the spacing of outer doublets around the missing outer doublet was also altered (Fig. 3I). Other abnormal axoneme structures included the loss of one outer doublet and one microtubule of another outer doublet (7×2 + 1×1 + 2) and the loss of two outer doublets and one microtubule from the central pair (7×2 + 1) (Fig. 3I). These results suggest an essential role for TbBLD10 in axoneme assembly.

### Depletion of TbPOC11 inhibits pBB biogenesis and flagellum assembly.

**FIG 4:**
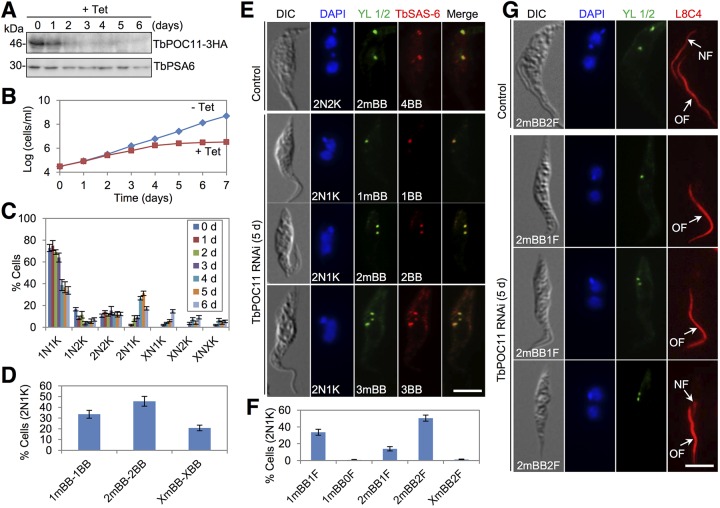
RNAi of TbPOC11 disrupts pro-basal body biogenesis and flagellum assembly. (A) Western blotting to monitor the protein level of TbPOC11. TbPOC11 was endogenously tagged with a triple-HA epitope in the TbPOC11 RNAi cell line. TbPSA6 served as the loading control. (B) TbPOC11 depletion inhibited cell proliferation. (C) Quantification of cells with different numbers of nuclei (N) and kinetoplasts (K) before and after TbPOC11 RNAi. A total of 200 cells were counted for each time point, and error bars indicate standard deviations calculated from three independent experiments. (D) Quantification of TbPOC11-deficient 2N1K cells with different numbers of mBBs and BBs (both mBBs and pBBs). A total of 200 cells were counted, and error bars indicate standard deviations from three independent experiments. (E) Coimmunostaining of cells with YL 1/2 to label mBBs and anti-TbSAS-6 antibody to label mBBs and pBBs. Bar, 5 µm. (F) Quantification of the numbers of mBBs and flagella in the 2N1K cells from TbPOC11 RNAi. A total of 200 cells were counted, and error bars indicate standard deviations calculated from three independent experiments. (G) Coimmunostaining of cells with YL 1/2 to label mBBs and L8C4 to label the flagella. NF, new flagellum; OF, old flagellum. Bar, 5 µm.

We investigated the role of TbPOC11, another conserved basal body protein that localizes to mBB and pBB (Fig. 1A). To examine the efficiency of RNAi, TbPOC11 was endogenously tagged with a triple-HA epitope in the TbPOC11 RNAi cell line, and Western blotting showed that upon RNAi induction TbPOC11-3HA was gradually depleted (Fig. 4A). TbPOC11 knockdown caused growth arrest after induction for 4 days (Fig. 4B) and led to the emergence of a larger population of 2N1K cells (~31% at day 5) and XN1K cells (X > 2, ~15% at day 6) and a smaller population of XN2K cells (X > 2, ~9% at day 6) and XNXK cells (X > 2, ~5% at day 6) (Fig. 4C). The accumulation of 2N1K and XN1K cells suggests defective kinetoplast segregation.

We investigated whether TbPOC11 depletion affected basal body duplication by immunofluorescence microscopy with anti-TbSAS-6 antibody and YL 1/2. We found that ~79% of the 2N1K cells contained either one mBB/one basal body (1mBB-1BB) or two mBBs/two basal bodies (2mBB-2BB) (Fig. 4D), suggesting that pBB biogenesis was inhibited. To examine whether flagellum formation was affected, we immunostained the cells with L8C4. Approximately 47% of the 2N1K cells contained only one flagellum, and the remaining 2N1K cells contained two flagella but the new flagellum in these cells was very short (Fig. 4F and G). TEM analysis of the flagellar axoneme in the RNAi cells showed that the axoneme was normal (data not shown). These results suggest that TbPOC11 depletion impairs new flagellum biogenesis without altering the axoneme structure.

### TbBBP65 is required for pBB biogenesis and axoneme assembly.

**FIG 5:**
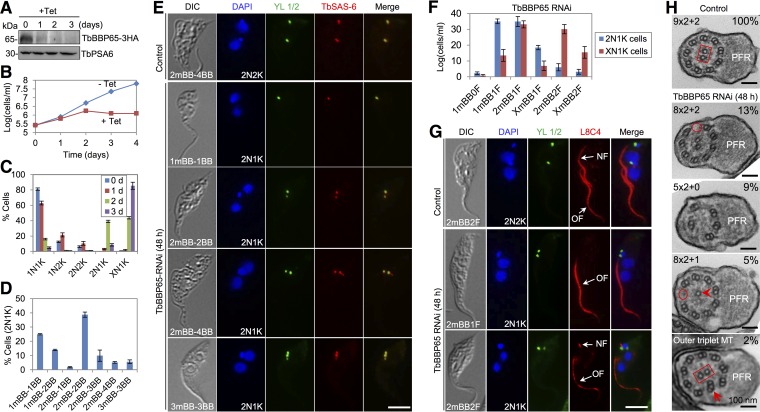
TbBBP65 is required for pro-basal body biogenesis and axoneme assembly. (A) Western blotting to examine the level of TbBBP65. TbBBP65 was endogenously tagged with a triple-HA epitope in the TbBBP65 RNAi cell line. TbPSA6 served as the loading control. (B) TbBBP65 RNAi inhibited cell proliferation. (C) Quantification of cells with different numbers of kinetoplasts (K) and nuclei (N) in control and TbBBP65 RNAi cells. A total of 200 cells were counted for each time point, and error bars indicate standard deviations calculated from three independent experiments. (D) Quantification of TbBBP65-deficient 2N1K cells with different numbers of mBBs and BBs (both mBBs and pBBs). A total of 200 cells were counted, and error bars indicate standard deviations calculated from three independent experiments. (E) Coimmunostaining of cells with YL 1/2 to label mBBs and with anti-TbSAS-6 antibody to label mBBs and pBBs. Bar, 5 µm. (F) Quantification of TbBBP65-deficient 2N1K cells and XN1K (X > 2) cells with different numbers of mBBs and flagella. A total of 200 cells were counted for each cell type, and error bars indicate standard deviations calculated from three independent experiments. (G) Immunostaining of cells with YL 1/2 to label mBBs and with L8C4 to label the flagella. Bar, 5 µm. (H) Morphology of flagellar axonemes in control and TbBBP65 RNAi cells. The red oval outlines a missing outer microtubule doublet in TbBBP65 RNAi cells. The red open arrowhead indicates a central microtubule singlet in a TbBBP65 RNAi cell. The red arrow indicates an outer microtubule triplet in a TbBBP65 RNAi cell. The red rectangle outlines the central microtubule pair in a control cell and a TbBBP65 RNAi cell. Note the orientation of the central pair in the RNAi cell was altered. Bar, 100 nm.

TbBBP65 RNAi caused rapid depletion of TbBBP65 protein, which was endogenously tagged with a triple-HA epitope in the RNAi cell line, from day 1 of RNAi (Fig. 5A) and resulted in growth arrest from day 2 of RNAi (Fig. 5B). After RNAi for 2 days, 2N1K cells increased to ~40% of the total cell population and XN1K (X > 2) cells increased to ~43% of the population (Fig. 5C). The XN1K cells further increased to ~85% of the total population after RNAi for 3 days (Fig. 5C). Immunofluorescence microscopy with anti-TbSAS-6 antibody and YL 1/2 showed that the majority (~80%) of the 2N1K cells contained either one basal body or two basal bodies (Fig. 5D and E), indicating that formation of pBB was impaired.

To explore the potential defects in flagellum assembly by TbBBP65 RNAi, we immunostained the flagellum with L8C4. In 2N1K cells, the majority (~88%) of them contained a single flagellum and the rest of them contained a short, new flagellum and a full-length old flagellum (Fig. 5F and G). In XN1K cells, ~54% of them contained a single flagellum and the rest of them contained a short, new flagellum and a full-length old flagellum (Fig. 5F). TbBBP65 RNAi also produced small-sized 1N1K cells without a flagellum or with a short flagellum (Fig. S6A, B, and C), which were produced by division of the 2N2K cells without a flagellum or with a short, new flagellum (Fig. S6D). These results suggest that new flagellum assembly is impaired by TbBBP65 depletion.

10.1128/mBio.02120-16.6 FIGURE S6  TbBBP65 RNAi produced 1N1K cells with a short flagellum or no flagellum. (A) Immunostaining of 1N1K cells from control and TbBBP65 RNAi (48 h) with L8C4 and YL 1/2. Bar, 5 µm. (B) Quantification of 1N1K cells with different numbers of mature basal bodies and flagella from control and TbBBP65 RNAi (48 h). mBB, mature basal body; sF, short flagellum. Error bars indicate standard deviations. (C) Measurement of flagellar length and cell length of the 1N1K cells from control and TbBBP65 RNAi (48 h) cells. About 75 cells from each cell line were measured and plotted. Those circled within the dotted oval indicate the TbBBP65 RNAi cells with a short flagellum. (D) Scanning electron microscopy analysis of control and TbBBP65 RNAi (48 h) cells. Panel a shows a control cell with a single flagellum. Panel b shows a control cell with two full-length flagella. Panel c shows a TbBBP65 RNAi cell without a flagellum. Panel d shows a TbBBP65 RNAi cell with a short, detached flagellum. Panel e shows a TbBBP65 RNAi cell with a full-length old flagellum and a short, new flagellum. Panel f shows a dividing TbBBP65 RNAi cell with a full-length old flagellum and a short, new flagellum. Bars, 5 µm. Download Figure S6, PDF file, 0.3 MB. Copyright © 2017 Dang et al. 2017 Dang et al. This content is distributed under the terms of the Creative Commons Attribution 4.0 International license.

TEM showed that ~29% of the flagellar axoneme exhibited an abnormal structure. The majority of the abnormal axoneme lost one outer doublet (Fig. 5H, red circle), and in some of these axonemes one microtubule of the central pair was also lost (Fig. 5H, open arrowhead). Other abnormal axonemes either lost four outer doublets and the central pair (5×2 + 0) or contained an outer triplet (Fig. 5H, red arrow) and a misoriented central pair (Fig. 5H, red rectangle). These results suggest that TbBBP65 is required for axoneme assembly.

### TbBBP46 is involved in basal body separation.

**FIG 6:**
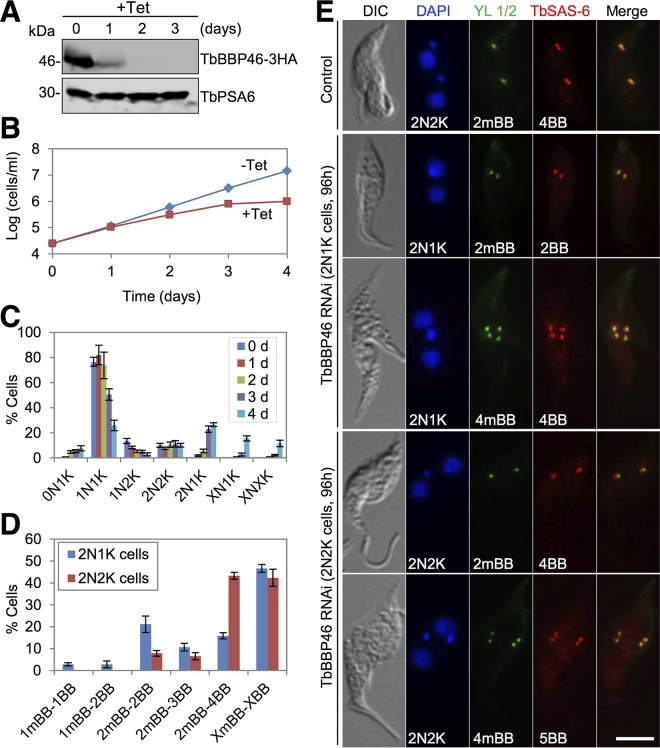
TbBBP46 is required for basal body separation. (A) Western blotting to examine the level of TbBBP46 before and after RNAi induction. TbBBP46 was endogenously tagged with a triple HA epitope in cells harboring the TbBBP46 RNAi construct. TbBBP46-3HA was detected by anti-HA antibody. TbPSA6 served as the loading control. (B) RNAi of TbBBP46 inhibited cell proliferation. (C) Quantification of cells with different numbers of kinetoplasts (K) and nuclei (N) before and after TbBBP46 RNAi. A total of 200 cells were counted for each time point, and error bars indicate standard deviations calculated from three independent experiments. (D) Effect of TbBBP65 RNAi on basal body duplication/separation. Shown is the quantification of the TbBBP46-deficient 2N1K and 2N2K cells with different numbers of mature basal bodies (mBBs) and basal bodies (both mBBs and pBBs). A total of 200 cells were counted, and error bars indicate standard deviations from three independent experiments. (E) Coimmunostaining of control and TbBBP46 RNAi cells with YL 1/2 antibody to label the mature basal body (mBBs) and with anti-TbSAS-6 antibody to label both the mature basal body and pro-basal body. Bar, 5 µm.

A number of proteins localized to mBB (Fig. 1B), and one of them, TbBBP46, was functionally characterized. Knockdown of TbBBP46, which was endogenously tagged with a triple-HA epitope, was confirmed by Western blotting with anti-HA antibody (Fig. 6A). TbBBP46 depletion resulted in growth inhibition after RNAi for 3 days (Fig. 6B) and resulted in the emergence of ~27% 2N1K cells and ~15% XN1K (X > 2) cells (Fig. 6C). There was also an accumulation of ~11% XNXK (X>2) cells (Fig. 6C), but the multiple kinetoplasts were not well segregated (data not shown). These results suggested that kinetoplast segregation was defective.

To examine whether TbBBP46 depletion disrupted basal body duplication or separation, we immunostained the cells with anti-TbSAS-6 antibody and YL 1/2. In the 2N1K cells, although ~21% of them contained two mBBs/two basal bodies (2mBB-2BB), ~16% of them contained two mBBs/four basal bodies (2mBB-4BB) and ~46% of them contained multiple (>2) mBBs/multiple (>2) basal bodies (XmBB-XBB, X > 2) (Fig. 6D and E). These basal bodies all clustered around the single kinetoplast (Fig. 6E), indicating that basal body separation was defective. In the 2N2K cells, ~43% of them contained two mBBs/four basal bodies (2mBB-4BB), and ~42% of them contained multiple (>2) mBBs/multiple (>2) basal bodies (XmBB-XBB, X > 2) (Fig. 6D and E). These basal bodies were all positioned between the two segregated nuclei (Fig. 6E), in contrast to the control 2N2K cells, in which one mBB/pBB pair migrated to the posterior region of the cell (Fig. 6E), indicating that basal body separation in the 2N2K cells was impaired. As a consequence, the two kinetoplasts in these 2N2K cells were also not far separated as in the control cells (Fig. 6E). Together, these results suggest an essential role of TbBBP46 in basal body separation.

### TbCEP57 is required for basal body separation.

**FIG 7:**
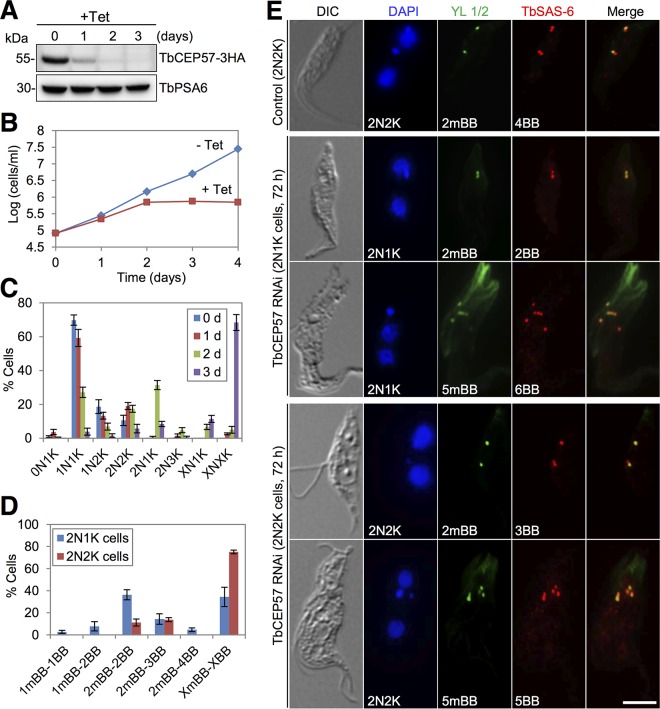
TbCEP57 is required for basal body separation and flagellum assembly. (A) Western blotting to monitor the protein level of TbCEP57 before and after TbCEP57 RNAi. TbCEP57 was endogenously tagged with a triple-HA epitope in cells harboring the TbCEP57 RNAi construct. TbPSA6 served as the loading control. (B) TbCEP57 is essential for cell proliferation. (C) Quantification of cells with different numbers of kinetoplasts (K) and nuclei (N) before and after TbCEP57 RNAi. A total of 200 cells were counted for each time point, and error bars indicate standard deviations calculated from three independent experiments. (D) Quantification of cells with different numbers of mature basal bodies (mBBs) and basal bodies (both mBBs and pBBs) in TbCEP57-depleted 2N1K and 2N2K cells. A total of 200 cells were counted for each cell type, and error bars indicate standard deviations calculated from three independent experiments. (E) Immunostaining of control and TbCEP57 RNAi cells with YL 1/2 antibody to label the mature basal body and with anti-TbSAS-6 antibody to label both the mature basal body and pro-basal body. Bar, 5 µm.

The intriguing localization of several proteins between mBB and pBB led us to hypothesize that these proteins may be involved in basal body segregation. To test this hypothesis, TbCEP57 was chosen for functional characterization. Depletion of TbCEP57 protein, which was endogenously tagged with a triple-HA epitope, occurred after RNAi induction for 2 days (Fig. 7A) and caused growth arrest after 2 days and cell death after 4 days (Fig. 7B). TbCEP57 ablation resulted in the accumulation of ~31% 2N1K cells after 2 days and the emergence of ~11% XN1K (X > 2) cells and ~68% XNXK (X > 2) cells after 3 days (Fig. 7C), indicating defective kinetoplast segregation.

To examine whether basal body separation was disrupted by TbCEP57 depletion, we immunostained the cells with anti-TbSAS-6 antibody and YL 1/2. We found that ~36% of the 2N1K cells contained two mBBs/two basal bodies (2mBB-2BB), and ~34% of the 2N1K cells contained multiple (>2) mBBs/multiple (>2) basal bodies (XmBB-XBB, X > 2) (Fig. 7D and E). These multiple basal bodies clustered around the single kinetoplast (Fig. 7E), suggesting defective basal body separation. In 2N2K cells, however, the majority (~75%) of them contained multiple (>2) mBBs/multiple (>2) basal bodies (XmBB-XBB, X > 2) and the rest of them contained two mBBs and two to three basal bodies (Fig. 7D and E). Notably, the multiple basal bodies in these 2N2K cells were not far separated as in the control 2N2K cells (Fig. 7E), indicating defective basal body separation. Taken together, these results suggest that TbCEP57 is required for basal body separation.

## DISCUSSION

The duplication and segregation of the basal body, one of the essential MTOCs in *T. brucei*, likely constitutes the first cytoskeletal event of the *T. brucei* cell cycle and is known to be crucial for kinetoplast segregation, flagellum biogenesis, and cell division. Previous bioinformatics analyses identified a number of *T. brucei* homologs of conserved centriole/basal body protein homologs (3, 4). However, whether these homologs are bona fide basal body proteins in *T. brucei* and whether they play a conserved function remain unknown. In this paper, we identified 5 new centriole/basal body protein homologs and confirmed the localization of 11 centriole/basal body protein homologs (Fig. 1; see also Table S1 in the supplemental material), among which seven homologs localize to mBB and pBB, three homologs localize to the distal ends of mBB and pBB, and one homolog (TbCEP57) localizes between mBB and pBB (Table S1; Fig. 1). It should be noted that in a previous report, TbDIP13 was not detected at the basal body using anti-TbDIP13 antibody and epitope tagging (14). However, we found that TbDIP13, tagged with a C-terminal triple-HA epitope, localized to mBB and pBB, albeit it was also detected at the middle portion of the old flagellum attachment zone filament (Fig. S3). Together, these results demonstrated that these centriole/basal body protein homologs associate with the basal body in *T. brucei*.

As a key component of the ancestral centriole/basal body module, BLD10/CEP135 acts as a microtubule-binding protein to stabilize microtubules and controls the assembly of the central microtubule pair in the flagellar axoneme in *Drosophila melanogaster* sperm cells (20). Despite lacking the N-terminal conserved BLD10/CEP135 domain (Fig. S5A), TbBLD10 localizes to the basal body (Fig. 1) and plays an essential role in pBB biogenesis (Fig. 3), similar to the function of TbSAS-6 (11). Notably, depletion of TbBLD10 disrupted flagellar axoneme assembly, causing the loss of one outer doublet and the alteration of the orientation of the central pair in some axonemes and the loss of one of the two central microtubules in some other axonemes (Fig. 3I). Such defects are different from that caused by depletion of *Drosophila* BLD10, which disrupted the central pair of the sperm flagellum without affecting the outer doublets (20). These differential effects on the axoneme structure between *T. brucei* and *Drosophila* suggest likely distinct mechanisms in axoneme assembly between the two organisms.

Our results also demonstrated an essential involvement of POC11 in basal body duplication. POC11 was originally identified as a component of the human centrosome proteome (21, 22) and is evolutionarily conserved, but the function of POC11 has never been investigated. TbPOC11 localizes to mBB and pBB, where the protein appears to form a half-ring structure around the basal body cartwheel (Fig. 1A and 2A). Our RNAi data showed that TbPOC11 is required for pBB biogenesis, similar to the roles of TbBLD10 (Fig. 3) and TbSAS-6 (11). Furthermore, although TbPOC11 depletion also inhibited flagellum elongation (Fig. 4F and G), the flagellar axoneme structure was not altered, in contrast to TbBLD10 RNAi (Fig. 3) and TbSAS-6 RNAi (11). These results suggest that TbPOC11 may regulate pBB biogenesis through a mechanism distinct from that of TbBLD10 and TbSAS-6.

Our work on TbCEP57 uncovered its novel function in controlling basal body separation in *T. brucei* (Fig. 7D and E). This unusual role of TbCEP57 is consistent with its unusual localization between mBB and pBB (Fig. 1F and 2A), which suggests that TbCEP57 may function as a basal body connector. Unlike TbCEP57, however, the CEP57 homologs in *Xenopus laevis* and humans localize to centrosomes and kinetochores and are required for spindle assembly and kinetochore-microtubule attachment (23, 24). Moreover, the human CEP57 homolog also functions as a NEDD1-binding pericentriolar material component to maintain spindle pole integrity (25). In contrast, TbCEP57 was not detected at the spindle poles and kinetochores (Fig. S3), and depletion of TbCEP57 caused the accumulation of multinucleated cells (Fig. 7C), suggesting that TbCEP57 is not required for nuclear division in *T. brucei*.

The identification of 25 trypanosome-specific basal body proteins (Table S1) suggests an unusual composition of the basal body proteome in *T. brucei*. The number of trypanosome-specific basal body proteins is comparable to that in *Tetrahymena* spp*.* (24 specific basal body proteins) (26) and is slightly more than half of the *Chlamydomonas*-specific basal body proteins (46 proteins) (22). However, given that the bait proteins used for BioID do not represent the proteins from different subdomains of the basal body, it is likely that our BioID analysis did not cover the entire basal body structure. Therefore, some other trypanosome-specific basal body proteins were likely not identified in our BioID experiments. Most of the 25 trypanosome-specific basal body proteins contain one or multiple coiled-coil motifs, which are also often found in the conserved centriole/basal body proteins (Table S1). These novel basal body proteins are located to different subdomains of the basal body (Fig. 1 and 2), suggesting potentially distinct functions. This notion was supported by the functional analysis of TbBBP46 and TbBBP65, which exhibited different localizations in the basal body (Fig. 1) and play distinct roles in basal body duplication and separation (Fig. 5 and 6). It should be noted that the defects caused by TbBBP65 depletion resemble those of TbSAS-6 RNAi (11) and TbBLD10 RNAi (Fig. 3; Fig. S5), both of which inhibited pBB biogenesis and disrupted the flagellar axoneme. Therefore, TbBBP65 may cooperate with the cartwheel components TbSAS-6 and TbBLD10 to regulate pBB biogenesis. Intriguingly, TbBBP65, as well as TbBBP72, forms a ring-like structure surrounding the basal body cartwheel (Fig. 2A). It is likely that TbBBP65 and TbBBP72 may function to stabilize the outer wall of the basal body barrel during basal body biogenesis.

In summary, this work identified novel regulators of basal body duplication and separation and revealed a new function of TbCEP57 in basal body separation. The identification and validation of 36 basal body proteins laid the foundation for understanding the assembly process of the basal body and the regulation of basal body duplication and separation. Importantly, some of the trypanosome-specific basal body proteins could serve as novel drug targets for chemotherapeutic intervention.

## MATERIALS AND METHODS

### Trypanosome cell culture.

The 427 strain of procyclic-form *T. brucei* was cultured in SDM-79 medium containing 10% heat-inactivated fetal bovine serum (Atlanta Biologicals, Inc.) at 27°C. The 29-13 strain of *T. brucei* was grown in SDM-79 medium supplemented with 10% fetal bovine serum, 15 µg/ml G418, and 50 µg/ml hygromycin.

### Proximity-dependent biotin identification.

The full-length coding sequences of TbSAS-6, TbCEP57, TbPOC11, and TbBBP46 were cloned into the pLew100-BirA*-HA vector (12), and the resulting plasmids were linearized with NotI and transfected into the 29-13 strain. Successful transfectants were selected with 2.5 µg/ml phleomycin and cloned by limiting dilution in a 96-well plate. Expression of BirA*-HA fusion proteins was induced with 0.1 µg/ml tetracycline for 24 h before adding 50 µM biotin and incubating for another 24 h.

Affinity purification of biotinylated proteins and mass spectrometric analysis of peptides were performed exactly as described in previous publications (12, 27, 28). Purified proteins were digested with trypsin and analyzed on an LTQ Orbitrap XL mass spectrometer (Thermo-Fisher Scientific) interfaced with an Eksgent Nano-LC 2D Plus chipLC system (Eksigent Technologies). Raw mass spectrometry data were searched against the *T. brucei* genome database by using the Mascot search engine.

### RNA interference.

To generate the TbBLD10, TbBBP65, TbCEP57, TbBBP46, and TbPOC11 RNAi cell lines, a DNA fragment corresponding to the coding region of each gene was cloned into the pZJM vector (29). Primer sequences are listed in Table S2. The resulting plasmids were linearized by NotI and electroporated into the 29-13 strain. Transfectants were selected with 2.5 µg/ml phleomycin and cloned by limiting dilution. RNAi was induced with 1.0 µg/ml tetracycline.

10.1128/mBio.02120-16.8 TABLE S2  List of primers for epitope tagging of basal body proteins (lowercase letters indicate sequences overlapping with the vectors). Download Table S2, XLSX file, 0.02 MB. Copyright © 2017 Dang et al. 2017 Dang et al. This content is distributed under the terms of the Creative Commons Attribution 4.0 International license.

### Endogenous epitope tagging of proteins.

Endogenous tagging of proteins with a triple-HA epitope at the C terminus was carried out using the PCR-based one-step approach (30), except for TbBLD10, which was tagged using the pC-TbBLD10-3HA-PAC vector. Primer sequences are listed in Table S2. PCR was performed with long primers, and PCR fragments were purified from the agarose gels and transfected into the 427 cell line or the respective RNAi cell line. Transfectants were selected with 1 µg/ml puromycin and cloned by limiting dilution. Correct epitope tagging at the endogenous locus was confirmed by sequencing, and expression of epitope-tagged proteins was verified by Western blotting with anti-HA antibody (1:1,000 dilution; Sigma-Aldrich).

### Purification of recombinant TbBLD10 protein and antibody production.

A 1,062-bp fragment corresponding to the C-terminal coding region (amino acids 401 to 754) of TbBLD10 was amplified from genomic DNA and cloned into the pET26 vector for expression of a hexahistidine-tagged TbBLD10 truncation protein in *Escherichia coli* strain BL21. The recombinant TbBLD10 truncation protein was purified under denaturing conditions by passing through a nickel column and used to immunize a rabbit to produce anti-TbBLD10 polyclonal antibody at Cocalico Biologicals, Inc. (Reamstown, PA). Crude antiserum was used directly for immunofluorescence microscopy and Western blotting (1:1,000 dilution).

### Immunofluorescence microscopy.

Cells were allowed to adhere to the coverslips were fixed with cold methanol (−20°C), rehydrated with phosphate-buffered saline (PBS) and then blocked in 3% bovine serum albumin in PBS. Immunostaining was performed by incubating the fixed cells with the primary antibody for 1 h at room temperature. The following primary antibodies were used: fluorescein isothiocyanate (FITC)-conjugated anti-HA monoclonal antibody (1:400 dilution; Sigma-Aldrich), L8C4 (anti-PFR2 monoclonal antibody [MAb]; 1:50 dilution) (31), anti-TbSAS-6 polyclonal antibody (1:400 dilution) (11), anti-TbBLD10 polyclonal antibody (1:400 dilution), and YL 1/2 (1:1,000 dilution; Millipore). The following secondary antibodies were used: Cy3-conjugated anti-mouse IgG (1:400 dilution; Sigma-Aldrich), FITC-conjugated anti-rat IgG (1:400 dilution; Sigma-Aldrich), and Cy3-conjugated anti-rabbit IgG (1:400 dilution; Sigma-Aldrich). Cells were washed with PBS, mounted with Vectashield mounting medium containing 4′,6-diamidino-2-phenylindole (Vector Labs), and imaged with an inverted fluorescence microscope (Olympus IX71) equipped with a cooled charge-coupled-device (CCD) camera (model Orca-ER; Hamamatsu) and a PlanApo N 60×, 1.42-numeric aperture differential inference contrast objective. Images were acquired using the Slidebook software (version 5; Intelligent Imaging Innovations).

### Transmission electron microscopy.

Preparation of thin sections of trypanosome cells for transmission electron microscopy was carried out according to methods described in our previous publication (32). Briefly, cells were fixed in glutaraldehyde, treated with Millonig’s buffer, and incubated with 2% OsO_4_. Cells were then dehydrated by treating with a serial concentration of ethanol solutions and embedded in resin. The 120-nm thin sections were cut using a Leica Ultracut-R microtome and a diamond knife (Daitome-US), placed on 150-mesh copper grids (Electron Microscopy Sciences, Hatfield, PA), and stained with 2% uranyl acetate. The thin sections were then rinsed with water and incubated with Reynold’s lead citrate. Grids were imaged using a JEOL 1400 TEM at 60 kV and captured with a Gatan CCD camera).

### Scanning electron microscopy.

Scanning electron microscopy was performed as described in our previous publications (27, 33). Briefly, cells were allowed to settle onto coverslips, fixed with 2.5% (vol/vol) glutaraldehyde in PBS for 30 min at room temperature, and then dehydrated in ethanol. After critical point drying, samples were coated with a 5-nm metal film (Pt:Pd at 80:20; Ted Pella Inc.) using a sputter coater (Cressington Sputter Coater 208 HR; Ted Pella Inc.) and imaged using Nova NanoSEM 230 (FEI).

### 3D-SIM superresolution microscopy.

Cells on the coverslips were coimmunostained with FITC-conjugated anti-HA monoclonal antibody and anti-TbSAS-6 polyclonal antibody. After thorough washing with PBS, cells were incubated with Alexa Fluor594-conjugated anti-rabbit IgG. Slides were examined under a DeltaVision OMX v 4 Blaze microscope (Applied Precision, GE Healthcare) to view the high-resolution localization patterns according to published procedures (34). The imaging data were then subjected to SI reconstruction and Image Registration using DeltaVision softWoRx software.
