# Supplementary material for: Proximity Interactions among Basal Body Components in Trypanosoma brucei Identify Novel Regulators of Basal Body Biogenesis and Inheritance
Source: mBio. 2017 Jan 3;8(1):e02120-16. doi: 10.1128/mBio.02120-16 (PMC5210500; doi:10.1128/mBio.02120-16)
Supplement: TABLE S1 [file mbo006163130st1.pdf]

Table S1: Conserved and novel basal body proteins in *T. brucei*

Protein name	Gene ID	Discovery methods	M.M. (kDa)	Motifs	Localization
TbCEP19	Tb927.10.10690	HsCEP19 homolog	43.2	1 x CC	N/A
TbCEP44	Tb927.7.6840	HsCEP44 homolog	19.7	1 x CC	N/A
TbCEP57	Tb927.10.5990	HsCEP57 homolog	50.8	3 x CC; 2 x DUF	Between pBB and mBB
TbCEP76	Tb927.11.8860	HsCEP76 homolog	70.9	1 x CEP76 C2	Distal ends of pBB and mBB
TbCEP120A	Tb927.7.6250	HsCEP120 homolog	49.0	4 x CC	pBB and mBB
TbCEP120B	Tb927.11.8920	HsCEP120 homolog	63.4	5 x CC	pBB and mBB
TbCEP164A	Tb927.5.2440	HsCEP164 homolog	130.9	8 x CC; 1 x WW	Distal ends of mBB
TbCEP164B	Tb927.11.11650	HsCEP164 homolog	111.2	8 x CC; 1 x WW	Distal ends of mBB
TbCEP164C	Tb927.1.3560	HsCEP164 homolog		4 x CC; 1 x WW	N/A
TbPOC1	Tb927.10.2860	HsPOC1 homolog	84.3	7 x WD40; 1 x CC	pBB and mBB
TbPOC5	Tb927.10.7600	HsPOC5 homolog	41.0	1 x CC	pBB and mBB
TbPOC11	Tb927.11.6560	CrPOC11 homolog	46.3	4 x CC	pBB and mBB
TbDIP13	Tb927.10.14110	DIP13/NA14 homolog	13.2	1 x CC	pBB and mBB
TbBLD10	Tb927.11.13700	CrBLD10 homolog	85.8	7 x CC	pBB and mBB
TbBBP38	Tb927.10.4210	Epitope tagging	38.2	1 x CC	mBB
TbBBP41	Tb927.3.4400	TbSAS-6 BioID	41.3	5 x CC	pBB and mBB
TbBBP46	Tb927.8.4210	TbCEP57 BioID	46.1	8 x WD40	mBB
TbBBP52	Tb927.10.14520	Epitope tagging	51.6	1 x CC	Distal ends of pBB and mBB
TbBBP58	Tb927.8.4580	TbPOC11 BioID	58.1	5 x CC; 1 x TPH	pBB, mBB and flagellum
TbBBP59	Tb927.10.350	TbBBP87 paralog	58.5	Protein kinase	pBB and mBB
TbBBP60	Tb927.8.2590	TbPOC11 & TbCEP57 BioID	58.5	1 x CC	mBB
TbBBP65	Tb927.5.4340	TbPOC11 & TbCEP57 BioID	65.5	3 x CC	pBB and mBB
TbBBP68	Tb927.10.3140	Epitope tagging	68.5	3 x CC; 6 x LRR	pBB
TbBBP69	Tb927.11.2700	Epitope tagging	69.2	3 x CC	Between pBB and mBB
TbBBP72	Tb927.10.15190	TbPOC11 & TbCEP57 BioID	72.7	1 x CC	Distal ends of pBB and mBB
TbBBP86	Tb927.10.5910	TbPOC11 & TbCEP57 BioID	85.8	3 x CC	pBB, mBB and bilobe
TbBBP87	Tb927.7.3880	TbCEP57 BioID	87.1	Protein kinase	Distal ends of pBB and mBB
TbBBP96	Tb927.3.5450	Epitope tagging	95.9	1 x CC	pBB and mBB
TbBBP109	Tb927.1.1100	TbPOC11 BioID	108.8	3 x CC; 1 x AIP3	pBB and mBB
TbBBP110	Tb927.10.12950	TbPOC11 & TbCEP57 BioID	109.2	12 x CC; 3 x IncA	pBB and mBB
TbBBP119	Tb927.11.15070	TbPOC11 & TbBBP46 BioID	119.2	None	Proximal ends of pBB and mBB
TbBBP120	Tb927.10.12870	TbPOC11 & TbCEP57 BioID	118.8	1 x CC; 1 x β-helix	pBB, mBB and new FAZ tip
TbBBP122	Tb927.8.6490	TbCEP57 BioID	119.4	Protein kinase; 2 x LRR	pBB and mBB
TbBBP135	Tb927.5.3280	TbCEP57 BioID	133.8	5 x CC; 2 x Transmembrane	pBB
TbBBP136	Tb927.3.5160	TbPOC11 & TbCEP57 BioID	134.6	None	mBB
TbBBP248	Tb927.7.7400	TbPOC11 & TbBBP46 BioID	248.0	9 x CC; 12 x Cornifin	Between pBB and mBB
TbBBP268	Tb927.10.10280	TbPOC11 & TbBBP46 BioID	267.6	1 x CC	pBB and mBB
TbBBP270	Tb927.11.15450	TbCEP57 BioID	269.6	31 x CC	Distal ends of pBB and mBB
TbBBP590	Tb927.11.10660	TbCEP57 BioID	590.1	31 x CC; 8 x ISXO2-like transposase; 2 x IncA	mBB
