# Supplementary material for: Proximity Interactions among Basal Body Components in Trypanosoma brucei Identify Novel Regulators of Basal Body Biogenesis and Inheritance
Source: mBio. 2017 Jan 3;8(1):e02120-16. doi: 10.1128/mBio.02120-16 (PMC5210500; doi:10.1128/mBio.02120-16)
Supplement: FIGURE S1 [file mbo006163130sf1.pdf]

Figure S1

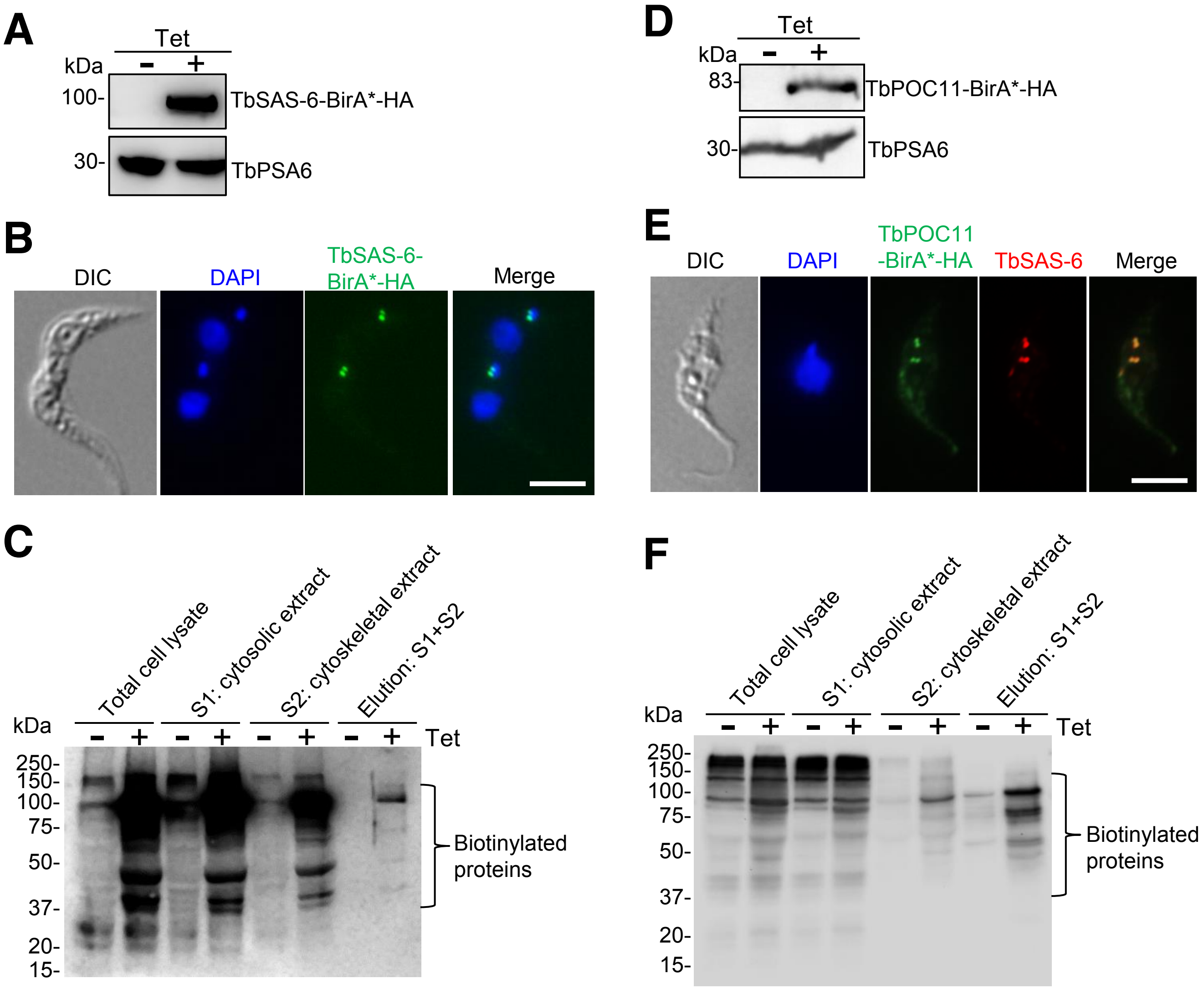
A
Tet
kDa
-
+
100-
TbSAS-6-BirA*-HA
30-
TbPSA6
B
DIC
DAPI
TbSAS-6-BirA*-HA
Merge
C
Total cell lysate
S1: cytosolic extract
S2: cytoskeletal extract
Elution: S1+S2
kDa
- + - + - + - +
Tet
250-
150-
100-
75-
50-
37-
20-
15-
Biotinylated proteins
D
Tet
kDa
-
+
83-
TbPOC11-BirA*-HA
30-
TbPSA6
E
DIC
DAPI
TbPOC11-BirA*-HA
TbSAS-6
Merge
F
Total cell lysate
S1: cytosolic extract
S2: cytoskeletal extract
Elution: S1+S2
kDa
- + - + - + - +
Tet
250-
150-
100-
75-
50-
37-
20-
15-
Biotinylated proteins

Figure S1 (continued)

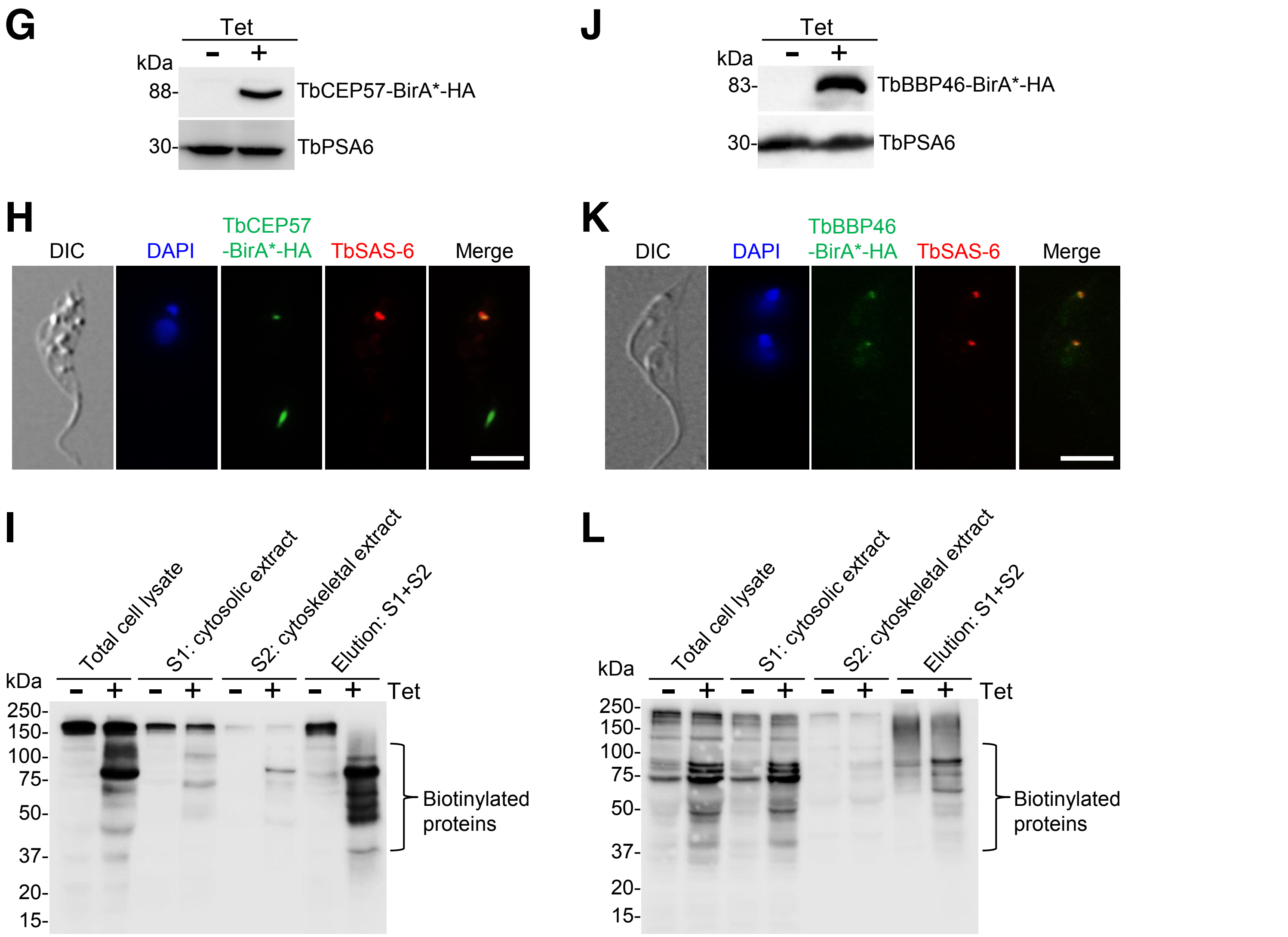

Figure S1. Identification of binding partners and near neighbors of TbSAS-6, TbPOC11, TbCEP57 and TbBBP46 by BioID. (**A, D, G, J**). Western blotting to detect the expression of BirA*-3HA-fused TbSAS-6 (**A**), TbPOC11 (**D**), TbCEP57 (**G**) and TbBBP46 (**J**). TbPSA6 served as the loading control. (**B, E, H, K**). Immunofluorescence microscopy to examine the localization of BirA*-3HA-fused TbSAS-6 (**B**), TbPOC11 (**E**), TbCEP57 (**H**) and TbBBP46 (**K**). Scale bar: 5 µm. (**C, F, I, L**). Affinity purification of biotinylated proteins from cells expressing BirA*-3HA-fused TbSAS-6 (**C**), TbPOC11 (**F**), TbCEP57 (**I**) and TbBBP46 (**L**). The non-induced cells served as the control.
