# Supplementary material for: Proximity Interactions among Basal Body Components in Trypanosoma brucei Identify Novel Regulators of Basal Body Biogenesis and Inheritance
Source: mBio. 2017 Jan 3;8(1):e02120-16. doi: 10.1128/mBio.02120-16 (PMC5210500; doi:10.1128/mBio.02120-16)
Supplement: FIGURE S2 [file mbo006163130sf2.pdf]

Figure S2

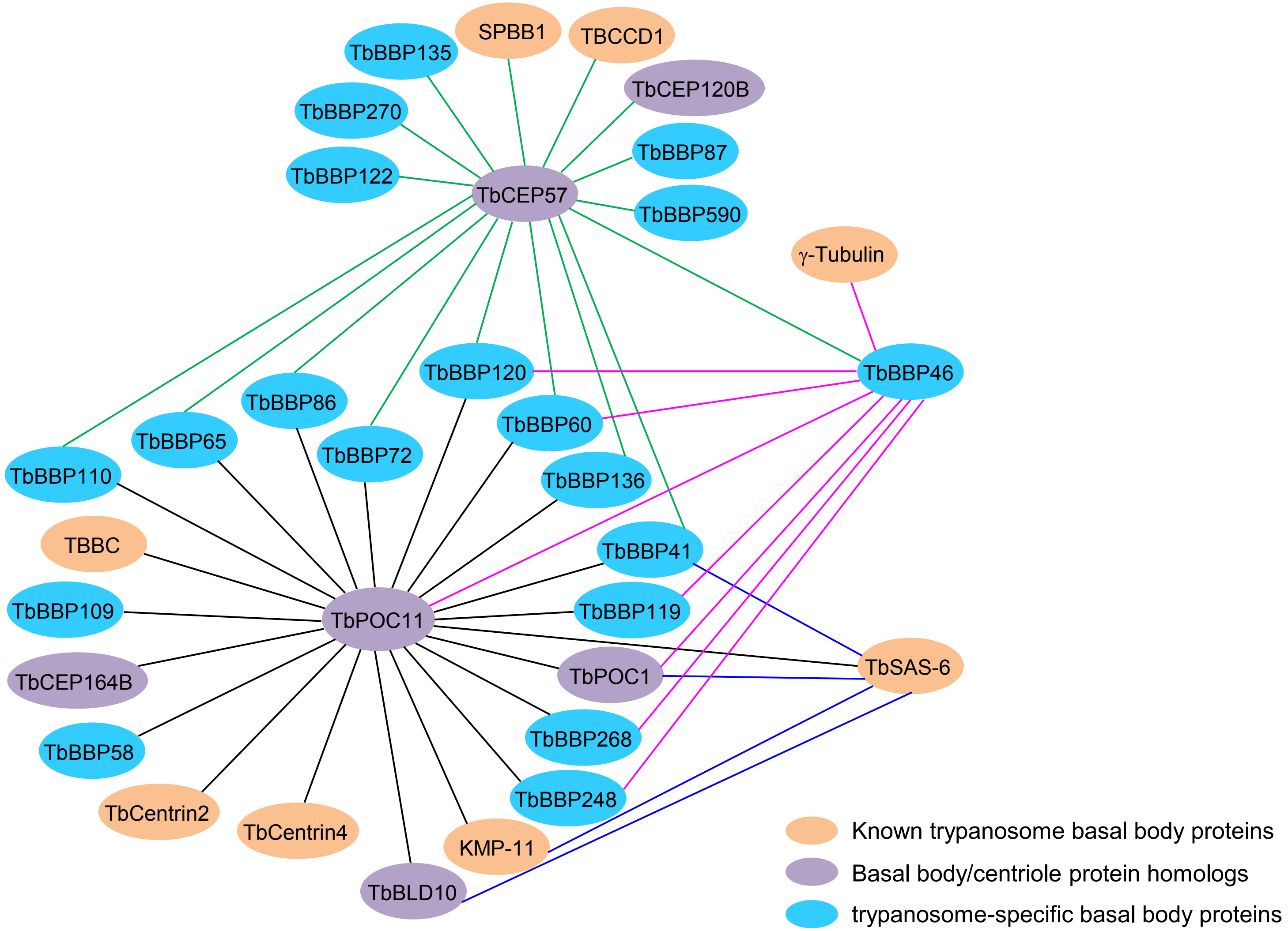

Figure S2. Proximity-based interaction map of *T. brucei* basal body proteins. BioID was carried out with TbSAS-6, TbPOC11, TbCEP57 and TbBBP46 as baits. Blue lines indicate the proximity interactions detected with TbSAS-6 BioID, black lines for the proximity interactions detected by TbPOC11 BioID, green lines for the proximity interactions detected by TbCEP57 BioID, and pink lines for the proximity interactions detected by TbBBP46 BioID.
