# Supplementary material for: Proximity Interactions among Basal Body Components in Trypanosoma brucei Identify Novel Regulators of Basal Body Biogenesis and Inheritance
Source: mBio. 2017 Jan 3;8(1):e02120-16. doi: 10.1128/mBio.02120-16 (PMC5210500; doi:10.1128/mBio.02120-16)
Supplement: FIGURE S3 [file mbo006163130sf3.pdf]

Figure S3

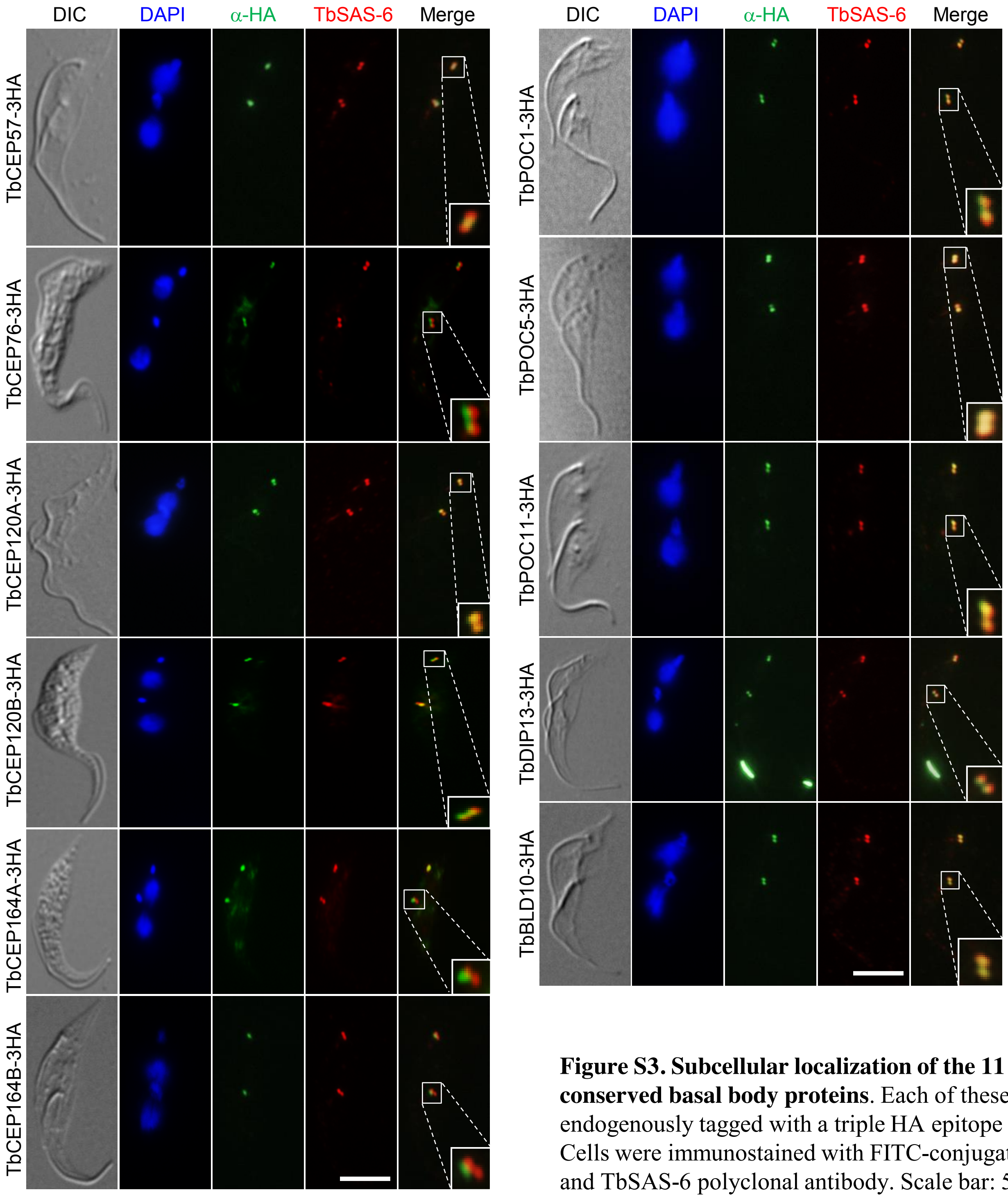

Figure S3. Subcellular localization of the 11 evolutionarily conserved basal body proteins. Each of these proteins was endogenously tagged with a triple HA epitope at the C-terminus. Cells were immunostained with FITC-conjugated anti-HA antibody and TbSAS-6 polyclonal antibody. Scale bar: 5 μm.
