# Supplementary material for: Proximity Interactions among Basal Body Components in Trypanosoma brucei Identify Novel Regulators of Basal Body Biogenesis and Inheritance
Source: mBio. 2017 Jan 3;8(1):e02120-16. doi: 10.1128/mBio.02120-16 (PMC5210500; doi:10.1128/mBio.02120-16)
Supplement: FIGURE S4 [file mbo006163130sf4.pdf]

Figure S4

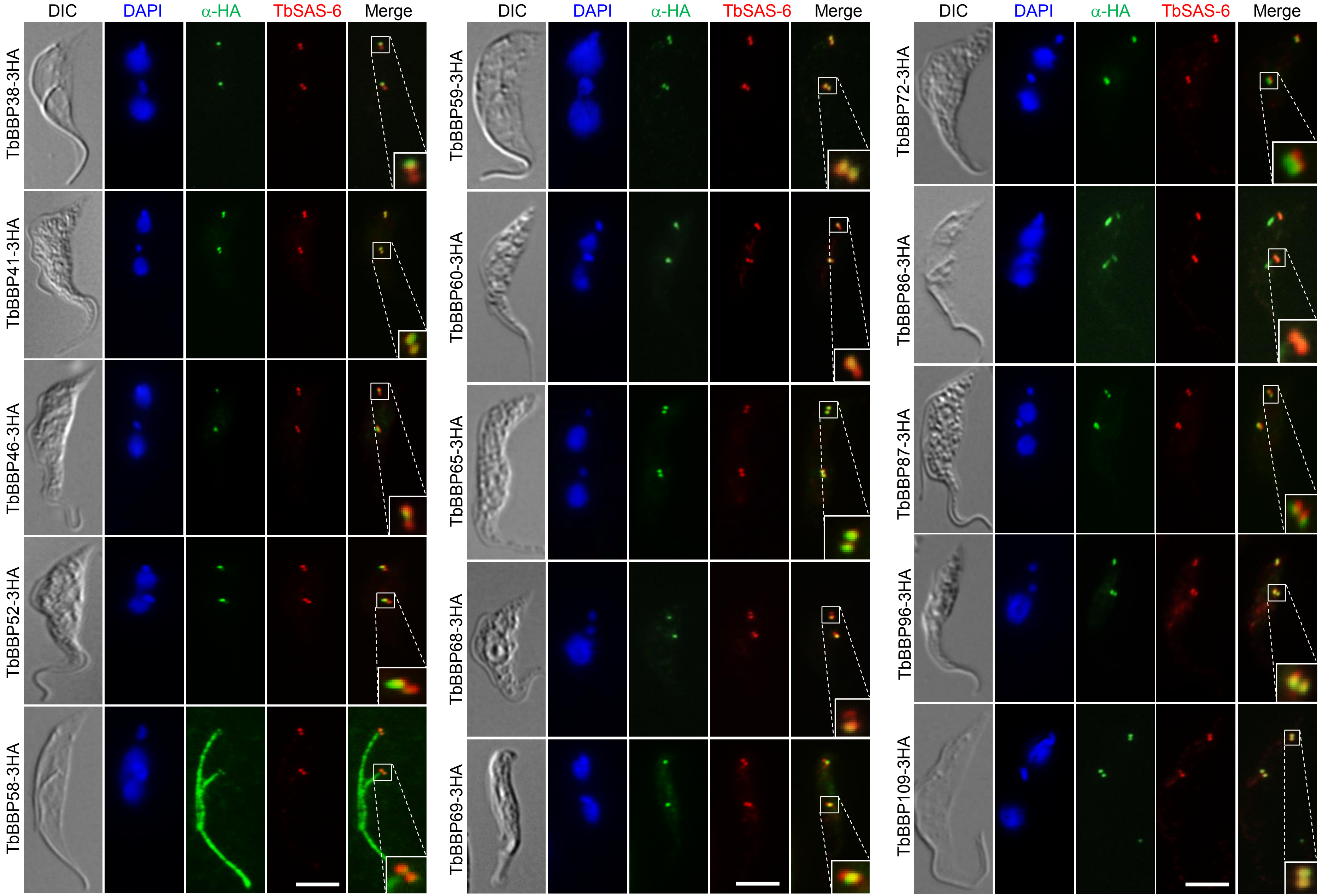
DIC
DAPI
α-HA
TbSAS-6
Merge
TbBBP38-3HA
TbBBP41-3HA
TbBBP46-3HA
TbBBP52-3HA
TbBBP58-3HA
TbBBP59-3HA
TbBBP60-3HA
TbBBP65-3HA
TbBBP68-3HA
TbBBP69-3HA
TbBBP72-3HA
TbBBP86-3HA
TbBBP87-3HA
TbBBP96-3HA
TbBBP109-3HA

Figure S4 (continued)

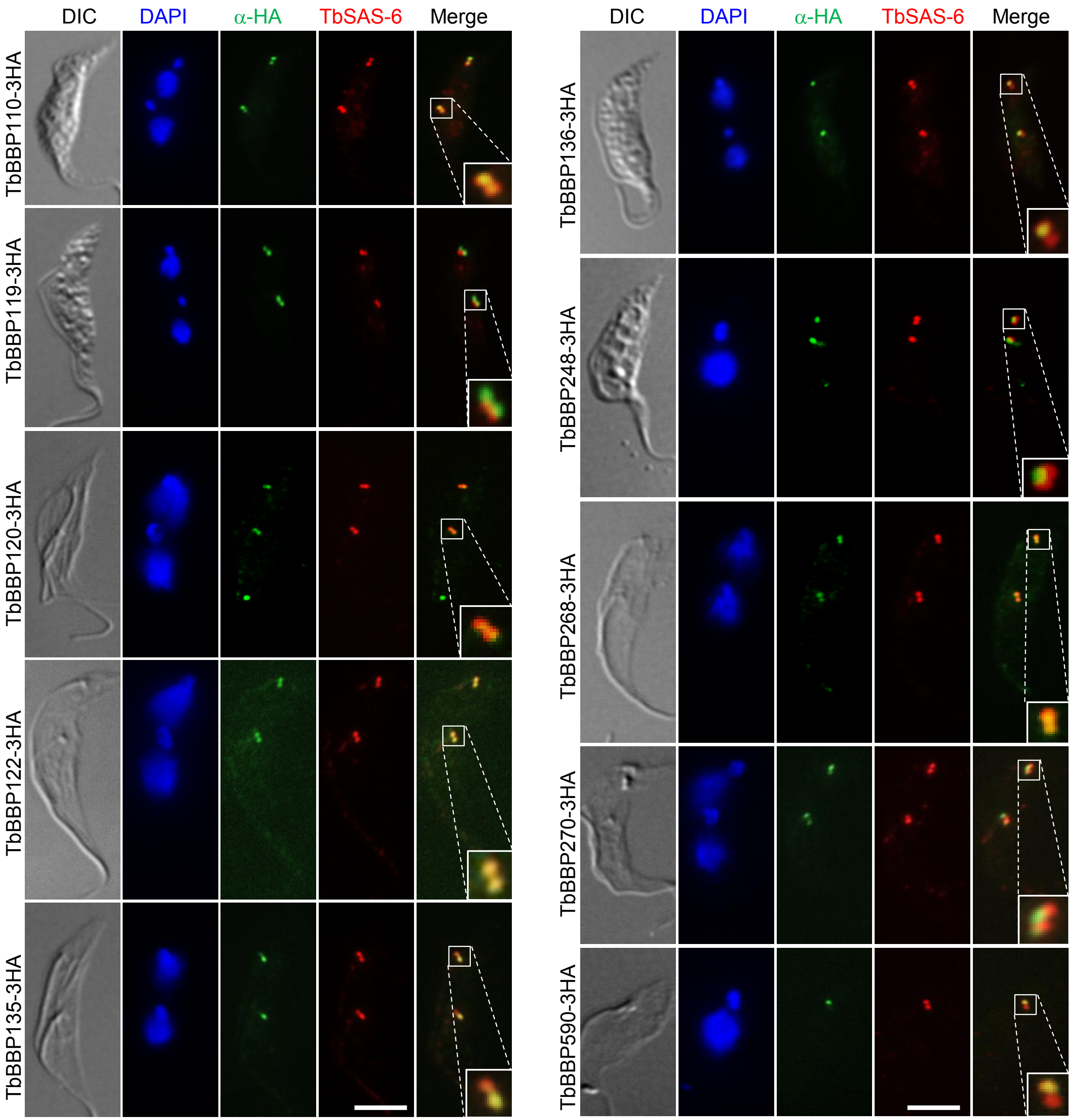

Figure S4. Subcellular localization of the 25 trypanosome-specific basal body proteins. Each of these proteins was endogenously tagged with a triple HA epitope at the C-terminus. Cells were immunostained with FITC-conjugated anti-HA antibody and TbSAS-6 polyclonal antibody. Scale bar: 5 µm.
