# Supplementary material for: Proximity Interactions among Basal Body Components in Trypanosoma brucei Identify Novel Regulators of Basal Body Biogenesis and Inheritance
Source: mBio. 2017 Jan 3;8(1):e02120-16. doi: 10.1128/mBio.02120-16 (PMC5210500; doi:10.1128/mBio.02120-16)
Supplement: FIGURE S5 [file mbo006163130sf5.pdf]

Figure S5

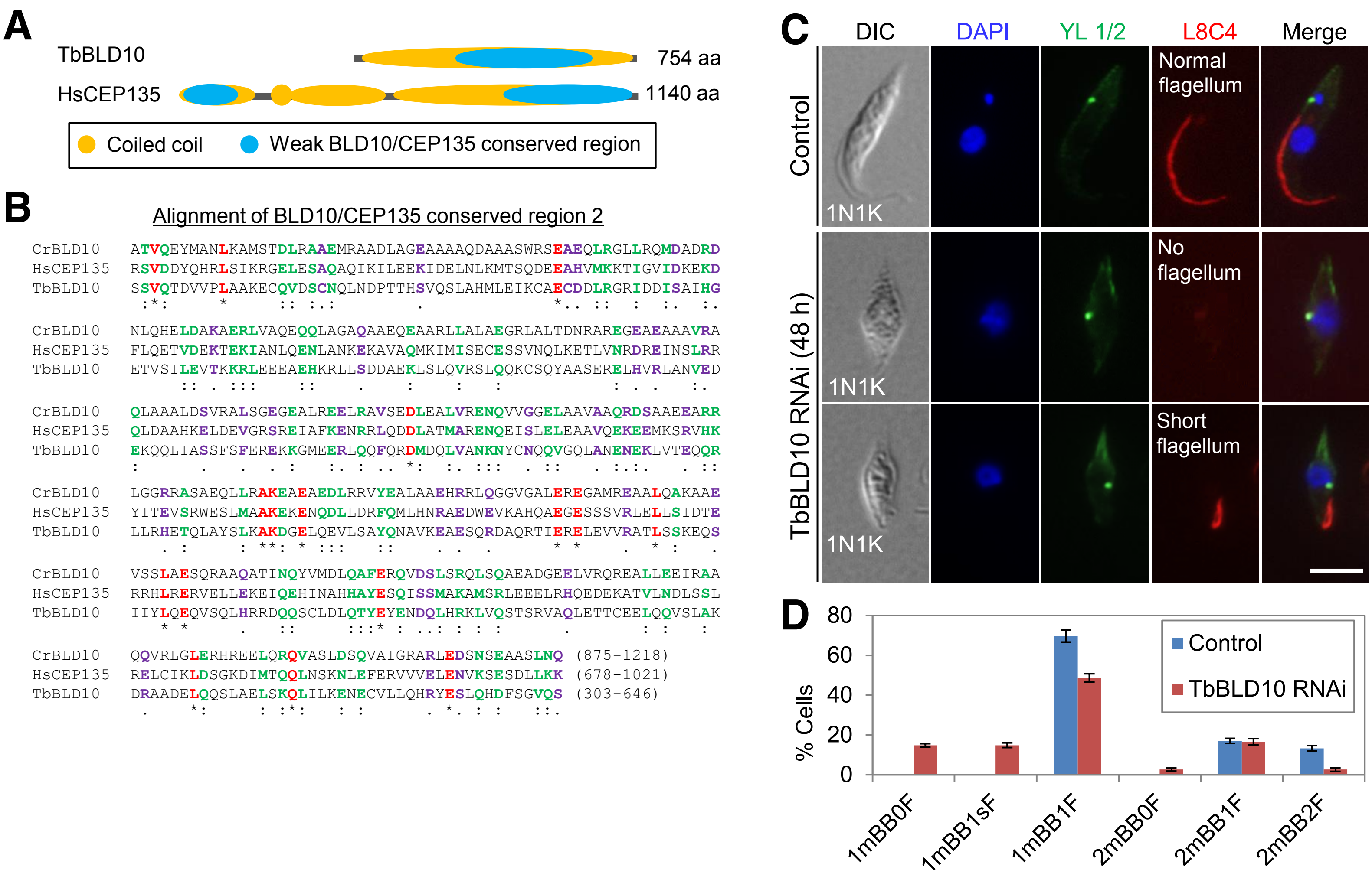

Figure S5. ***T. brucei*** **expresses a highly divergent BLD10 homolog.** (**A**). Schematic drawing of the conserved domains in TbBLD10 and the human BLD10 homolog HsCEP135. (**B**). Sequence alignment of the BLD10/CEP135 conserved region among Chlamydomonas reinhardtii BLD10 (CrBLD10), human CEP135, and TbBLD10. Identical residues are highlighted in red and indicated by asterisks (*), whereas homologous residues are highlighted in green and indicated by colons (:) or periods (.). (**C, D**) TbBLD10 RNAi produced 1N1K cells with a short flagellum or no flagellum**.** (**C**). Immunostaining of 1N1K cells from control and TbBLD10 RNAi (48 h) with L8C4 and YL 1/2. Scale bar: 5 µm. (**D**). Quantification of 1N1K cells with different numbers of mature basal body and flagellum from control and TbBLD10 RNAi (48 h). mBB, mature basal body; sF, short flagellum. Error bars indicate S.D.
