# Supplementary material for: Proximity Interactions among Basal Body Components in Trypanosoma brucei Identify Novel Regulators of Basal Body Biogenesis and Inheritance
Source: mBio. 2017 Jan 3;8(1):e02120-16. doi: 10.1128/mBio.02120-16 (PMC5210500; doi:10.1128/mBio.02120-16)
Supplement: FIGURE S6 [file mbo006163130sf6.pdf]

Figure S6

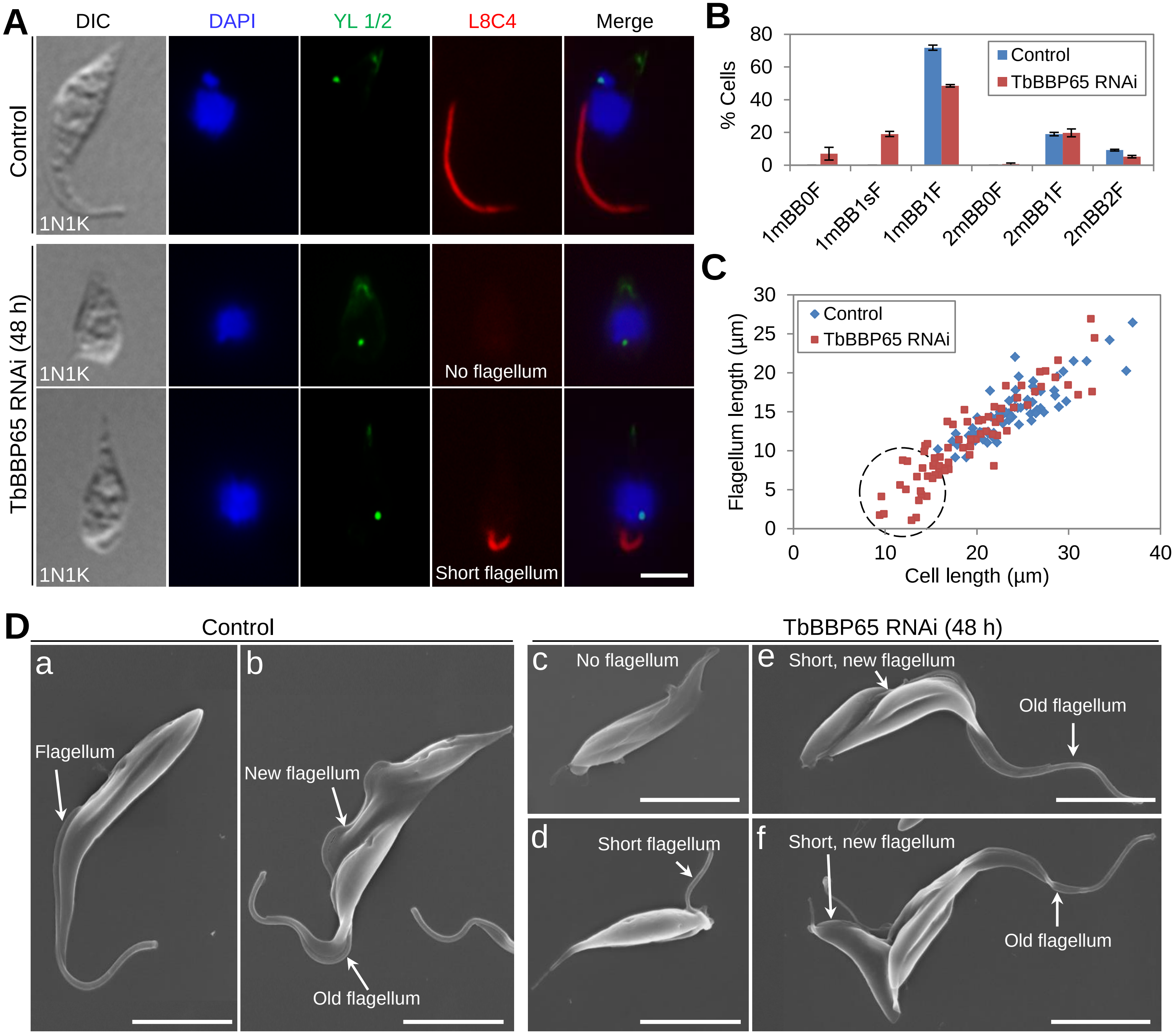

Figure S6. TbBBP65 RNAi produced 1N1K cells with a short flagellum or no flagellum. (**A**). Immunostaining of 1N1K cells from control and TbBBP65 RNAi (48 h) with L8C4 and YL 1/2. Scale bar: 5 µm. (**B**). Quantification of 1N1K cells with different numbers of mature basal body and flagellum from control and TbBBP65 RNAi (48 h). mBB, mature basal body; sF, short flagellum. Error bars indicate S.D. (**C**). Measurement of flagellum length and cell length of the 1N1K cells from control and TbBBP65 RNAi (48 h) cells. About 75 cells from each cell line were measured and plotted. Those circled within the dotted oval indicate the TbBBP65 RNAi cells with a short flagellum. (**D**). Scanning electron microscopy analysis of control and TbBBP65 RNAi (48 h) cells. Panel a shows a control cell with a single flagellum. Panel b shows a control cell with two full-length flagella. Panel c shows a TbBBP65 RNAi cell without flagellum. Panel d shows a TbBBP65 RNAi cell with a short, detached flagellum. Panel e shows a TbBBP65 RNAi cell with a full-length old flagellum and a short, new flagellum. Panel f shows a dividing TbBBP65 RNAi cell with a full-length old flagellum and a short, new flagellum. Scale bars: 5 µm.
